# Pneumonia*Paper read before the Atlanta Society of Medicine, February 4, 1896.

**Published:** 1896-03

**Authors:** J. C. Olmsted

**Affiliations:** Atlanta, Ga.


ATLANTA

Medical and Surgical Journal.

VOL. XIII. MARCH, 1896. No. 1.

LUTHER B. GRANDY, M.D., MANAGING EDITOR.

M. B. HUTCHINS, M.D., BUSINESS MANAGER.

COLLABORATORS:
H. V. M. MILLER, M.D., LL.D., A. W. CALHOUN, M.D., LL.D., VIRGIL O. HARDON, M.D., FLOYD W. McRAE, M.D., AND DUNBAR ROY, M.D.

### ORIGINAL COMMUNICATIONS.

### PNEUMONIA.*

BY J. C. OLMSTED, M.D.,
ATLANTA, GA.

*Definition.*—Before entering upon a discussion of the pathology of Pneumonia it would be well, I think, to define what is meant by the term "Pneumonia." By the term Pneumonia, then, I understand to be meant "Acute Lobar" or "Croupous" Pneumonia, as it is sometimes called by our German brethren, or "Pneumonitis," as it is frequently designated, an acute general disease characterized by an inflammation of the vesicular structure of the lungs with an exudation into the alveoli, or air-cells, which renders them impermeable to air, producing a condition called "hepatization"; a single lobe, the whole lung, or both lungs may be simultaneously involved. This definition, which is essentially that of Loomis, is sufficiently clear, and while free from some mooted points, I think expresses well the distinction between this form and the next most

*Paper read before the Atlanta Society of Medicine, February 4, 1896.

common form known as "Broncho" or "Catarrhal Pneumonia," whose pathology is essentially and widely different, as you will remember. Perhaps, as mirroring, in brief space, the current views of advanced medical authors and teachers a few more definitions may not be without interest.

Thus—Prof. Osler says (rather dogmatically, I think), for his definition, "Pneumonia,—an infectious disease, caused by the micrococcus lanceolatus (pneumococcus, diplococcus pneumoniæ), which excites a local inflammation in the lungs, and by its toxins constitutes disturbances of varying intensity. The fever terminates abruptly by crisis; secondary infective processes are common." (In regard to certain portions of this definition I would reply: "highly probable but not proven!" Again, we find in the last edition of "Quain's Dictionary of Medicine" the following more conservative definition: "Pneumonia, an acute disease characterized clinically by sudden onset of severe febrile symptoms, cough, expectoration, and dyspnea, by the physical signs of pulmonary consolidation, and by a rapid abatement of the general symptoms between the fourth and tenth days. Anatomically, it is characterized by an acute inflammation of the lung tissue and by the accumulation of the inflammatory products within the air-cells, which products consist mainly of a fibrinous exudation and leucocytes."

Again I cannot overlook a criticism of that eminently wise and conservatively progressive teacher and writer, the late Dr. Austin Flint. In defining the disease pneumonia, under the head of "Acute Lobar Pneumonia," "Croupous Pneumonia," or "Pneumonitis" (terms which he accepted as conforming to general usage), he yet protests in these words: "These terms are incorrect; all imply that the disease is a purely local inflammation, whereas there are sufficient grounds for regarding it as an essential fever!" Oh! prophetic and wise seer, who in these words has anticipated what will doubtless become the settled faith of modern medical science! These definitions, I believe, fairly express the present attitude of medical opinion, as far as brief definition can. Before passing to the most interesting field of inquiry, the etiology of the disease, a brief summary of the morbid anatomy might be best. Since the time of Laennec pathologists have recognized three "stages" of the inflamed lung, viz.: "Engorgement," "Red Hepatization," and "Gray Hepatization." In the stage of engorgement, the lung is deep red in color, firmer to the touch, and more solid. On section the surface is bathed with blood and serum, it still crepitates (not so distinctly as healthy lung), excised portions float, air-cells can be insufflated from the bronchus, microscopic examination shows great distention of capillary vessels, alveolar epithelium is swollen, and the air-cells are occupied by a variable number of blood corpuscles and detached alveolar cells.

In second stage, "Red Hepatization," lung tissue is solid, firm, airless. On section surface is dry, reddish-brown in color, and very friable, tearing very easily, in marked contrast to healthy lung tissue. Careful inspection shows a granular surface, the granulations representing fibrinous plugs filling air-cells. Smaller branches often contain fibrinous plugs. In the meshes of this coagulated fibrin are found red-blood corpuscles, polynuclear leucocytes, and alveolar epithelium. Alveolar walls are infiltrated with leucocytes; diplococci, staphylococci, and streptococci are also found. In the third stage, "Gray Hepatization," the tissue is changed to a grayish white color, due to the air-cells being densely packed with leucocytes. In this, known as the "Stage of Resolution," disintegration of cells and detritus by fatty degeneration takes place, enabling absorption and elimination to occur. When this course is interrupted, a condition of "purulent infiltration" exists, with the formation of abcesses, such as are found post mortem. At times all three stages of the pneumonic process may be seen in one lung, and the passage from engorgement to "Red" and "Gray Hepatization" distinctly traced. The bronchial mucous membrane is red, rarely swollen; fibrinous casts may be found in them. The bronchial glands are swollen and may be soft and pulpy. When the lung is superficially involved, the pleural surface shows a thin sheet of exudate, giving turbidity to that membrane.

*Lesions of other Organs.*—The heart is distended with firm tenacious coagula. Right chamber is markedly distended. The spleen is enlarged, kidneys show parenchymatous swelling and turbidity of cortex, with at times interstitial changes. Pericarditis is not infrequent; five in one hundred cases (Osler). Endocarditis more frequent; sixteen in one hundred cases (Osler). Meningitis is not infrequent. Croupous or diphtheritic inflammation of the colon and other parts has been observed. The liver shows parenchymatous changes and engorgement of veins.

We will now consider the *etiology* of this, which is one of the most widespread of acute diseases.

We find that of predisposing causes age ranks first. Prevailing at all ages, there are yet three marked periods: early childhood, then from twenty to forty years, and lastly in old age, of which it is the especial enemy; and according to Loomis, after the age of sixty, nine-tenths of the deaths from acute diseases are due to pneumonia. While catarrhal pneumonia is more common than croupous pneumonia in young children, yet the latter is also found in them, and, according to Loomis, it is five times more frequent in the first two years of existence than in the following eighteen. Males are more frequently affected than females, as being subject to more exposure, for in advanced age, when the conditions of living are more nearly the same, the proportion is about equal. Dwellers in cities, and especially in crowded and unhygienic surroundings, those exposed to hardship and the vicissitudes of weather, are more liable to the disease.

Alcoholism is a most potent predisposing cause. Chronic diseases, such as diabetes, Bright's and others, previous attacks of pneumonia; in short, all debilitating influences exercise a marked effect as "predisposing causes." Pneumonia following blows on the chest, and other traumatism, is known as "Contusion, or Traumatic Pneumonia," and the injury producing it is simply a predisposing cause.

No acute disease recurs with such frequency as Pneumonia. Twenty-nine recurrent attacks are mentioned by Loomis, and ten by Osler, in one individual. The influence of cold as the most potent cause of this disease, and its prevalence during the winter months, has been urged by the supporters of the "purely local inflammation" theory. Now, the disease is almost unknown in the Arctic regions, and statistics show that the continuously cold months give the lowest death-rate, while the spring months, with their sudden and marked changes of temperature, give the highest.

Thus, while not denying the profound effect of sudden chilling of the body, it appears that marked vicissitudes of weather are more active as a predisposing cause than the influence of mere cold. And again we find such diseases as cerebro-spinal meningitis, diphtheria, influenza, measles, etc., more prevalent in the winter months, and yet cold is not ascribed as their cause on that account. It is perhaps at this point that it may be well to consider the reasons for regarding Pneumonia as an infectious disease of the type of the essential fevers. The literature of the past ten years is richer in the development of facts and experimental investigation than all the conturies which have preceded; and the recent experiments, combined with modern statistics, the results of the microscope, and pathological histology, give Croupous Pneumonia a separate and distinct place in the list of pulmonary diseases. The old pathology, which assumed the disease to be a "*purely localized* inflammation of the lungs," and the constitutional symptoms as secondary to this. is no longer tenable. Pneumonia is now almost universally regarded as an infectious disease of the type of the essential fevers, with characteristic local manifestations and lesions in the lung. The disease appears to be caused by certain micro-organisms, or bacteria (more or less satisfactorily demonstrated to be always present in post mortem examinations), and the infection of the organism by germs which is favored by many different conditions, many of which have already been described under the head of "predisposing causes," and which may be summed up as, any or all causes which tend to lessen the vital forces and weaken the powers of resistance.

These germs, or bacteria, which appear to be innocuous under conditions of perfect health, develop and manifest their power when they find a proper soil (so to speak) prepared for them in the lessened vital resistance of the organism. Reasons for regarding the disease as an infectious one, resembling the essential fevers (and by the term infectious is not meant infection in the sense of contagion from individual to individual), may be briefly summed up as follows: First, like most infectious diseases, it runs a definite, self-limited course. Secondly, the relation between the inflammatory process in the lung and the constitutional symptoms is far from uniform. The fever and other signs of infection may, and do often, precede the local affection, and, as a rule, the fever vanishes before there has been demonstrable diminution of the pulmonary inflammation. Again, the constitutional symptoms may be of the severest type when the amount of lung tissue involved is small; and on the other hand, the course of the disease may be benign when an entire lobe or lung is consolidated. Such facts as these are not explained satisfactorily, on the theory that the general symptoms are secondary and dependent upon the local inflammation, for it is contrary to our experience in all other purely inflammatory processes. Thirdly, another proof of the infectious nature of the disease, is that at times it prevails in epidemic form, numerous examples from prisons and barracks having been given, and one remarkable epidemic at Middleburgh, a town in England, where in 1888, out of a population of 70,000, there were 369 deaths. Some village epidemics have indeed been remarkable, resembling contagious diseases in their wide range. Again, it acts primarily upon the nervous system, shown frequently at the outset in young children by convulsions, and in the aged by delirium and coma passing into death. The delirium often present, and the disturbance of the vaso-motor system is very marked. Like other infectious diseases, it has its complications, such as peri- and endocarditis, and derangement of the kidneys, liver, and other organs. Fourth. Fraenkel, and others following him, claims to have found a micro-organism, known as the "Diplococcus of Fraenkel,"* to be most commonly present in the inflamed lung."

---

*SPECIAL NOTE.—The diplococcus pneumonia of Fraenkel is identical with the micrococcus which Sternberg and Pasteur found in the saliva of certain persons, and which produces septicemia in the rabbit. This is the most constant organism found in pneumonia, occurring also in the secondary processes of the diseases such as pleursy, endocarditis pericarditis and meningitis. In ten cases observed in the Pathological Laboratory of "Johns Hopkins University," it was present in all. In six of them as pure cultures, and in four associated with pus organisms. It is present also in the sputa of pneumonic cases. It is elliptical in form, lance-shaped, occurring in pairs, hence the name "Diplococcus." It may attack other parts, such as the meninges in cerebro-spinal meningitis, independent of inflammation of the lung. Most interesting and suggestive, are recent experiments in Leyden's Clinic, made by the Klemperer Brothers, on the production of immunity and upon the cure of pneumonia. Immunity was readily obtained in animals by either subcutaneous or intravenous injections of quantities of the filtered bouillon cultures, or glycerine extract. Immunity rarely lasted more than six months, and was transmitted to offspring born within this period. More interesting yet were their experiments on the cure of the artificially produced disease. Fluids and serum of an animal rendered immune not only produced immunity when injected into another animal, but actually cured the disease after injection had been in progress for some time. The temperature fell from 104° Fahrenheit and 105.6° Fahrenheit to normal in 24 hours

Friedlander also has his name associated with one of the bacteria. While again the streptococcus pyogenes, staphylococcus aureus and albus, only have been found. It is therefore evident that cases which do not depart clinically or anatomically from types of pneumonia may be associated with different species of organisms. The present views concerning the bacteriology of pneumonia may be summed up in the conclusions of Sturges and Coupland. (1) Pneumonia is a specific general disease of which lobar inflammation of the lung is the local manifestation. (2) The most constant cause of pneumonia is Fraenkel's diplococcus, but there are other viruses which may evoke it. (3) Chill and other causes, some more or less distinctly observed in their relation to the cause, are to be regarded as only secondary or predisposing causes. It would then appear, I think, that while the bulk of evidence points to bacteriological origin of the disease, it is but fair to say of the particular bacilli described, that their rôle as the causative agents of this disease has not up to the present date reached the position of a clear and undoubted demonstration; and we must yet hold the special forms, under judgment, subject to further experimentation and verification, while holding to the infectious and general, or constitutional nature of this disease in contradistinction to the old "localized inflammation" theory. Nor is it unimportant that we should make this distinction, and have clear views as to its being a general constitutional disease, for the practical question of treatment is pregnant with results either good or bad, according to the conceptions of the disease which hold. Remembering that the disease is self-limited and that its tendency is usually toward recovery,

---

They believe that the pneumococcus produces a poisonous albumin ("Pneumotoxin"), which, introduced into the circulation of an animal, causes elevation of the temperature and subsequent production in the body of a substance "anti-pneumotoxin," which possesses the power of neutralizing the poisonous albumin, which is formed by the bacilli. In man they hold that in pneumonia there is a constant absorption of the poisonous albumin produced by the bacteria in the lungs. This continues until the same antidotal substance is produced in the circulation that has been seen to accrue experimentally. It is then that the "crisis" occurs. Bacteria are not destroyed, nor is their power to produce the poisonous albumin lessened, but the latter is neutralized by the third factor, or "anti-pneumotoxin" element. He demonstrated that the serum of the blood after "crisis" in patients, contained this antitoxic substance, which cured infected animals when injected. They also reduced the "crisis" in six patients, by injecting the serum of convalescent patients, which contained this substance, or "antitoxin." The general results of their experimental work have been confirmed by others, but up to the present date no satisfactory progress has been made in establishing a rational serum therapy for the disease in man.

that its chief danger is from heart failure and not apnea, our efforts will be directed to supporting the vital powers, and sustaining the heart, while abstaining from all debilitating measures and remedies which will depress its power. The heart, as you know, while suffering with the other tissues from the very high febrile temperature, has a burden put upon it of twofold character; it has to force blood through an obstructed lung and is obliged to increase its beats in order to aërate the blood in the limited portion it may be of unobstructed lung, this making up by rapid action or movement of the blood, for the lost extent of lung surface for oxygenation under the old plan of cutting short the inflammation; the lancet, tartar emetic, and later veratrum were invoked as "specifics." It is not for me to here discuss the question of treatment; that has been left for abler hands; but I would remark that the testimony from past experience in the treatment of pneumonia, and the discarding of these depressing agents, points to the practical abandonment of the old "localized inflammation" theory; whatever of utility they may have had in the past, they no longer possess.

As the great Trousseau (one of those dead but sceptered sovereigns who yet rule our spirits from their urns) once said:

"The medical or epidemic constitution changes at different periods." By "epidemic or medical constitution," he meant the reaction of a people to disease, and hence to medical remedies. Without going into his masterly disquisition upon this subject, I would say, that the generations which stood the heroic bleedings, drenchings with tartar emetic, and pint doses of veratrum, were not the same people in their constitutional condition, habits of living, etc., as the present generation of very restless individuals, all nervous system, for whom steam, at sixty miles an hour, is too slow traveling, and electricity the only tolerable medium for moving, seeing, and hearing! In conclusion, while awaiting further information as to the precise form of bacteria concerned in the etiology of this disease, I consider myself justified in regarding pneumonia as an acute infectious disease, an essential fever, whose peculiar lesions are found in the lung, as the distinctive lesions of typhoid fever are found in Peyer's patches of the small intestine.

